# ACE2 function in the pancreatic islet: Implications for relationship between SARS‐CoV‐2 and diabetes

**DOI:** 10.1111/apha.13733

**Published:** 2021-10-02

**Authors:** Bushra Memon, Essam M. Abdelalim

**Affiliations:** ^1^ College of Health and Life Sciences Hamad Bin Khalifa University (HBKU) Qatar Foundation Doha Qatar; ^2^ Diabetes Research Center Qatar Biomedical Research Institute (QBRI) Hamad Bin Khalifa University (HBKU) Qatar Foundation (QF) Doha Qatar

**Keywords:** angiotensin‐converting enzyme 2, beta cells, COVID‐19, glucose homoeostasis, insulin

## Abstract

The molecular link between SARS‐CoV‐2 infection and susceptibility is not well understood. Nonetheless, a bi‐directional relationship between SARS‐CoV‐2 and diabetes has been proposed. The angiotensin‐converting enzyme 2 (ACE2) is considered as the primary protein facilitating SARS‐CoV and SARS‐CoV‐2 attachment and entry into the host cells. Studies suggested that ACE2 is expressed in the endocrine cells of the pancreas including beta cells, in addition to the lungs and other organs; however, its expression in the islets, particularly beta cells, has been met with some contradiction. Importantly, ACE2 plays a crucial role in glucose homoeostasis and insulin secretion by regulating beta cell physiology. Given the ability of SARS‐CoV‐2 to infect human pluripotent stem cell‐derived pancreatic cells in vitro and the presence of SARS‐CoV‐2 in pancreatic samples from COVID‐19 patients strongly hints that SARS‐CoV‐2 can invade the pancreas and directly cause pancreatic injury and diabetes. However, more studies are required to dissect the underpinning molecular mechanisms triggered in SARS‐CoV‐2‐infected islets that lead to aggravation of diabetes. Regardless, it is important to understand the function of ACE2 in the pancreatic islets to design relevant therapeutic interventions in combatting the effects of SARS‐CoV‐2 on diabetes pathophysiology. Herein, we detail the function of ACE2 in pancreatic beta cells crucial for regulating insulin sensitivity, secretion, and glucose metabolism. Also, we discuss the potential role played by ACE2 in aiding SARS‐COV‐2 entry into the pancreas and the possibility of ACE2 cooperation with alternative entry factors as well as how that may be linked to diabetes pathogenesis.

## 1 INTRODUCTION

Coronavirus disease 2019 (COVID‐19), caused by severe acute respiratory syndrome coronavirus 2 (SARS‐CoV‐2), is a severe respiratory infectious disorder declared pandemic by the World Health Organization (WHO). Patients with diabetes and its associated metabolic disorders are highly susceptible to SARS‐CoV‐2 infection. In addition, compared with non‐diabetic, SARS‐COV‐2 infected patients with pre‐existing diabetes suffer from a more severe form of the disease, have a higher mortality rate and more incidence of multiple organ failure.^1^, ^2^ Underlying molecular mechanisms are not clearly understood; however, increased inflammatory cytokines and adipokines secreted in obese conditions, a weakened immunity, hypokalaemia, as well as lower physiological pH are postulated to increase the risk of infection.^3^ This leads to another important issue of debate: Can SARS‐COV‐2 cause diabetes and how? Interestingly, development of acute pancreatitis or pancreatic injury has also been reported during the course of infection.^4^, ^5^, ^6^, ^7^, ^8^, ^9^ Additionally, new onset hyperglycaemia and ketoacidosis are also increasingly observed in COVID‐19 patients with no previous history of type 2 diabetes (T2D), indicating metabolic misbalance.^10^, ^11^, ^12^ Increasing cases of new onset type 1 diabetes (T1D) have been reported in children with COVID‐19 infection.^13^ Of note, new onset insulin‐dependent diabetes has been reported in subjects with no serum antibodies against islet cells following SARS‐CoV‐2 infection, highlighting the case for virus‐induced pathogenesis of beta cell dysfunction.^14^, ^15^, ^16^ Whether SARS‐CoV‐2 can infect the pancreatic islets directly in humans resulting in islet cell apoptosis, dysregulated metabolism and pancreatitis is being explored. Of note, few groups have demonstrated the expression of SARS‐CoV‐2 in pancreatic tissue of COVID‐19 deceased patients in addition to the susceptibility of human islets to SARS‐CoV‐2 infection. Nonetheless, a bi‐directional relationship between COVID‐19 and diabetes is presumed with no causative molecular mechanisms discovered.

SARS‐CoV‐2 enters the human cells by binding to the angiotensin‐converting enzyme II (ACE2) receptor,^16^ a component of the renin–angiotensin system (RAS), which is expressed in lung cells as well as in different tissues.^17^, ^18^, ^19^ ACE2 cleaves Angiotensin II into Angiotensin (1‐7). Angiotensin (1‐7) binds to its receptor Mas whereas Angiotensin II binds to its receptors Angiotensin type I and II (ATR1 and ATR2), leading to antagonistic downstream processes. While RAS signalling and receptor ACE2 have been previously implicated in the pathogenesis of acute respiratory distress syndrome (ARDS) and severe acute respiratory syndrome (SARS), ACE2 receptor, in coordination with other proteins, also facilitates the invasion of SARS‐CoV‐2 in alveolar epithelial cells, alveolar macrophage, and pulmonary endothelium.^16^ The RAS plays an important role in pancreas biology and function.^20^ In humans, ACE2 expression has been demonstrated to be present in the pancreatic islets by some groups; however, others have shown contradictory results. ACE2 is expressed in the pancreas, specifically, beta cells, acinar and ductal cells as well as in the islet microvasculature and pericytes.^4^, ^21^ However, more recently, variation in the ACE2 localization within the islets was demonstrated in COVID‐19 deceased patients, indicating the differences in disease severity and outcome reported as well as contradiction amongst studies.^22^ Furthermore, it also highlights potential coordination of ACE2 with other surface receptors explored as entry factors for SARS‐CoV‐2 invasion, particularly in the pancreas. Nonetheless, several studies from rodent islet biology have given critical insights into importance of ACE2 protein signalling in controlling insulin secretion and hyperglycaemia. Understanding the role of ACE2 in the pancreatic islet, therefore, could help us uncover the crucial link between COVID‐19 infection and diabetes.

## 2 PHYSIOLOGICAL ROLE OF ACE2 AND ANGIOTENSIN (1‐7) IN PANCREATIC ISLETS

Local RAS in the pancreatic islet regulates glucose homoeostasis through key players like ACE2 and Angiotensin (1‐7)^20^ (Table 1). It is also known that certain components and genetic variants of the RAS components are associated with acute pancreatitis, such as ACE I, that results in lower ACE activity of hydrolyzing Angiotensin I to Angiotensin II, and Renin rs5707G, which is hypothesized to result in higher RAS activity.^23^ On the other hand, ACE2 deficiency can impact islet development and beta cell function (Table 1).^24^, ^25^ Within the pancreas, ACE2 is highly expressed in human insulin‐secreting beta cells compared with other islet cells, in addition to the pericytes or endothelial cells surrounding the islets.^21^, ^26^

**TABLE 1:** Role of different RAS components in pancreatic islets development and function

RAS component or signalling axis	Biological process	Function	References
Ang II	‐ Islet survival. ‐ Insulin secretion.	‐ Vasoconstriction of islet blood vessels. ‐ Inhibition of glucose‐stimulated insulin secretion	[27, 28]
Ang II/ AT2R	‐ Islet development	‐ Endocrine lineage commitment of pancreatic progenitors	[27]
Ang (1‐7)	‐ Islet survival in diabetes	‐ Prevents islet endothelial and endocrine cell apoptosis. ‐ Attenuates cellular stress and ‐ promotes glucose‐stimulated insulin secretion	[30, 31, 56]
Ang (1‐7)/ AKT/ eNOS/ NO	‐ Islet survival	‐ Vasodilation of islet endothelial cells	[31, 32]
Ang (1‐7)/ MAS	‐ Islet development ‐ Beta cell proliferation ‐ Glucose homoeostasis	‐ Regulates beta cell area and ratio within the islet ‐ Increases Ins+/Ki67+ proliferative beta cells ‐ Induces cAMP production in beta cells ‐ Increases expression of Pdx1, Ngn3, and Ins	[24, 56, 57]
Ang II/ ACE/ AT1R	‐ Cellular and oxidative stress	‐ ROS production by increasing NADPH oxidase activity	[38]
ACE2/ Ang (1‐7)/ MAS	‐ Insulin secretion ‐ Protect from oxidative stress. ‐ Glucose homoeostasis	‐ Improves calcium flux, insulin granule exocytosis, mitochondrial membrane potential in the presence of ROS, modulation of GAD67/ GABA signalling	[36, 42]
ACE2/ Ang (1‐7)	‐ Beta cell dedifferentiation	‐ Reverses Ins+/Oct4+ co‐expression in beta cells under high‐fat diet by increased islet microcirculation and VEGF expression as well as reduction of islet iNOS activity	[34, 55]
Ang (1‐7)/Ang (1‐2)/ Neprilysin/ GPRC6A	‐ Beta cell functionality	‐ Enhances insulin secretion	[37]
ACE2	‐ Mitochondrial metabolism in beta cells. ‐ Oxidative stress. ‐ Adaptive beta cell response under high‐fat conditions. ‐ Beta cell mass and islet size	‐ Upregulates mitochondrial genes and insulin secretion in the presence of ROS. ‐ Decreases NADPH oxidase activity and ROS production. ‐ Regulates the increase in beta cell mass and adaptive hyperinsulinemic response to high‐fat diet. ‐ Increases total insulin content in islets. ‐ Increases beta cell proliferation and prevents apoptosis.	[25, 33, 39, 40, 41, 43, 54, 55]

Interestingly, the receptors for Angiotensin II, AT1R and AT2R, are expressed in pancreatic progenitor cells isolated from the human foetal pancreas as well as in beta‐like cells.^27^ AT2R, localized to the nuclei, is co‐expressed with Neurogenin 3 (NGN3)‐positive cells in differentiating pancreatic progenitors. ATR2 depletion prevents further differentiation of pancreatic progenitor cells into islet‐like clusters.^27^ AT2R has been found to be co‐expressed with PDX1 and INSULIN in the islet‐like clusters and is primarily cytoplasmic. Of note, other RAS components like angiotensinogen and renin expression have been detected in islet‐like clusters only.^27^ Angiotensin II levels increased during the course of differentiation of foetal pancreatic progenitor to islet‐like clusters, where it upregulates PDX1 and INS expression. This indicates that angiotensin II, through its receptor AT2R, mediates differentiation of pancreatic progenitor cells isolated from human foetal pancreas towards endocrine lineage.^27^ Therefore, extensive studies are still needed to understand the importance of ACE2‐Angiotensin II‐Angiotensin (1‐7) system in the human pancreas. Nevertheless, investigations in rodents have shed light on the functioning of ACE2 in the pancreas and in diabetes.

Angiotensin II (Ang II) is predominantly known for causing the contraction of blood vessels in the pancreas, as well as inhibits both first and second phase of glucose‐stimulated insulin secretion (GSIS) and islet cell survival (Table 1).^28^ Furthermore, angiotensin receptor inhibition causes vasodilation, improves islet blood flow and improves GSIS.^28^, ^29^ On the other hand, the cleaved product of Ang II, angiotensin (1‐7) (Ang (1‐7)), causes vasodilation by improving intra‐islet vessel density in T2D rats as well as increases total insulin content in islets and the first‐phase secretion of insulin by beta cells.^30^ Also, Ang (1‐7) treatment decreases apoptotic islet cells in these T2D rats.^30^ Additionally, Ang (1‐7) prevents palmitate‐induced apoptosis in islet endothelial cells^31^ and activates Akt/eNOS/NO pathway in MS‐1 murine pancreatic endothelial cell line by enhancing AKT and eNOS phosphorylation and ultimately NO production (Table 1).^31^, ^32^ Co‐culturing of Ang (1‐7)‐treated MS‐1 endothelial cells with beta cells improves GSIS as well as attenuated palmitate‐induced beta cell apoptosis.^32^ In line with this data, ACE2 deficiency leads to decreased microvessel density and insulin content in islets of ACE2 knock‐out (KO) mice under high‐fat diet, further hindering GSIS.^33^ These studies indicate that Angiotensin II‐Angiotensin (1‐7) level dynamics regulated by ACE2 controls islet microcirculation and thereby affecting islet survival and function.

Interestingly, ACE2 is highly expressed in alpha cells in mice, which is dissimilar to its expression pattern in rats.^34^, ^35^ Mice fed with the standard diet have ~65% of Ace2^+^/Gcg^+^ cells in the islet in comparison to ~19% Ace2^+^/Ins^+^ cells.^34^ Additionally, the G‐protein‐coupled receptor for Ang (1‐7) which is the enzymatic product of ACE2, MAS, is localized to insulin‐expressing cells.^34^ This indicates that ACE2 may play an important role in paracrine signalling in islet endocrine cells. Moreover, Ang (1‐7) increases expression of Pdx1, Insulin and Ngn3 in mice embryonic pancreatic explants, which is inhibited by a MAS receptor antagonist.^24^ MAS receptor inhibitor also alters beta cell: alpha cell ratio, as well as decreases beta cell area per islet.^24^ This highlights that Ang (1‐7)/MAS signalling regulates islet development (Figure 2). Pancreatic explants treated with the MAS receptor inhibitor decreases numbers of Ins+/Ki67+ cells indicating a decline in beta cell proliferation (Table 1).^24^ One mechanism by which ACE2/Ang (1‐7)/MAS pathway maintains glucose homoeostasis is by modulating the GAD67/GABA signalling in pancreatic islets.^36^ Interestingly, Ang (1‐7) enhances insulin secretion in mice through the Ang (1‐7)/Ang (1‐2)/GPRC6A axis as well, which is mediated through the proteolytic action of another peptidase called Neprilysin that generates the smaller peptide Ang (1‐2) from Ang (1‐7) (Table 1).^37^

Ang II is known for triggering the generation of reactive oxygen species (ROS) such as superoxide and hydrogen peroxide. Ang II binds to its receptor AT1R and causes activation of Nox1 and Nox2 complexes and an increased NADPH oxidase activity, thereby augmenting ROS production.^38^ Oxidative stress and ROS production are harmful for islet function and hamper beta cell survival and insulin secretion. Notably, ACE2 aids in attenuating pancreatic oxidative stress and generation of superoxide radicals and ROS induced by Ang II in multiple cell types.^39^, ^40^, ^41^ ACE2 gene deletion increases NADPH oxidase activity in mice resulting in higher oxidative stress, which is further increased upon Ang II treatment. These effects are reversed upon ACE2 overexpression in these mice.^41^ Therefore, it is likely that ACE2 plays a protective role by checking oxidative stress induction by lowering of Ang II levels as well as downregulation of overactive Ang II/ACE/ATR1 axis and reduction of NADPH oxidase activity (Table 1).^39^, ^41^

On the other hand, Ang (1‐7) that is formed as a result of cleavage of Ang II by ACE2, also reverses ROS production in INS‐1 β cell line and protect them from oxidative stress.^42^ Cells stimulated with hydrogen peroxide showed impaired insulin secretion that is improved by Ang (1‐7) pretreatment.^42^ Ang (1‐7) also improves peak calcium flux during release of insulin granules in the presence of ROS stimulators in INS‐1 β cells as well as restores mitochondrial membrane potential. Interestingly, inhibition of the MAS receptor blocks these protective effects of Ang (1‐7), indicating a potential role of ACE2/Ang (1‐7)/MAS axis in preventing oxidative stress (Table 1).^42^ Within the cell nucleus in the renal cortex, a ACE2‐Ang‐(1‐7)‐AT7R pathway has been identified that it prevents DNA damage by downregulating Ang II‐AT1R activity.^40^ Therefore, ACE2 and Ang (1‐7) may have synergistic effects on keeping oxidative stress in limits in pancreatic beta cells, as well as in other cell types (Figure 2).

In addition to oxidative damage, ACE2 plays a crucial role in mitochondrial metabolism in pancreatic beta cells. Overexpressing ACE2 in INS‐1 cells in the presence of ROS stimulators upregulates mitochondrial genes and attenuates the harmful effects of ROS on beta cell functionality by improving insulin secretion (Table 1).^43^ Since, treatment with Ang (1‐7) improves mitochondrial membrane potential in the presence of ROS stimulator in INS‐1 cells, it is likely that ACE2, through Ang (1‐7), maintains metabolic homoeostasis in pancreatic beta cells.

Although the role of local pancreatic RAS in islet function and diabetes has been widely studied in animal models, there are only a limited number of studies that evaluate its role in humans; thus, all results obtained from animal models cannot be extrapolated to humans due to the physiological differences.

## 3 REGULATION OF ACE2 TRANSCRIPTION AND ACTIVITY

**FIGURE 1:**
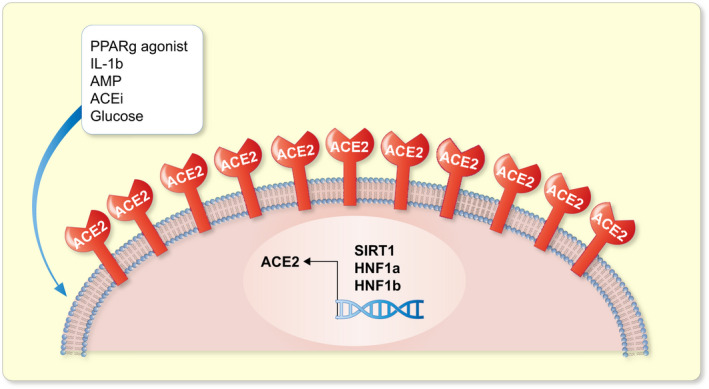
Factors regulating the expression and transcription of ACE2 receptor. Key transcription factors such as HNF1a, HNF1b and SIRT1 bind to the human ACE2 promoter and its mRNA expression. PPARg agonist can increase ACE2 protein expression in insulin‐sensitive tissues whereas high glucose exposure increases its enzyme activity. Interleukin 1 beta (IL‐1b) and AMP also induce ACE2 expression. Treatment with the ACE inhibitors (ACEi) increases ACE2 expression and activity thereby improving beta cell function

Given the beneficial role played by ACE2 in pancreatic development, metabolism and function, it is crucial to understand the regulatory pathways controlling ACE2 expression in order to exploit them for therapeutic use. Crucial beta cell transcription factors (TFs) and genes responsible for MODY3 and MODY5, such as HNF1A and HNF1B, have been identified to dose‐dependently enhance ACE2 transcript and protein expression as well as enzymatic activity by binding to the proximal region of the human ACE2 promoter.^44^, ^45^ Interestingly, in mice, a high‐fat diet and palmitatic acid treatment that increased free fatty acids led to cytoplasmic localization of HNF1A, excluded from the nucleus, in primary islet cells (Figure 1).^46^

Inflammatory stress, such as hypoxic conditions, Interleukin 1β and AICAR, an AMP mimic molecule, also induce ACE2 expression. The NAD+deacetylase SIRT1 binds to the ACE2 promoter and increases ACE2 transcription under energy stress (Figure 1). Interestingly, PPARγ agonists, such as pioglitazone and rosiglitazone can increase ACE2 protein expression in insulin‐sensitive tissues such as the skeletal muscle, adipose and liver.^47^ Short‐term exposure to high glucose in rat pancreatic beta cells BRIN‐BD11 also induces upregulation of the ACE2/Ang (1‐7)/Mas axis components as well as increases ACE2 enzymatic activity, thereby promoting GSIS.^48^ In addition to glycaemic levels, obesity also alters ACE2 expression thereby establishing a link between ACE2 levels and lipid metabolism. For example, obese mice models have been shown to have high ACE expression in the lung, which is further increased upon downregulation of sterol response element binding proteins 1 and 2 (SREBP) that control adipogenesis and lipid synthesis.^49^

Cell surface expression of ACE2 is regulated by a disintegrin and metalloproteinase 17 (ADAM17).^50^ ADAM17 cleaves catalytically active ACE2 bound at the cellular surface into the extracellular environment.^51^ Overexpression of ADAM17 increases shedding of ACE2 and decreases cellular bound ACE2 in mouse pancreatic islets.^51^ Interestingly, levels of ACE2 and ADAM17 mRNA and activity does not change in db/db mice nor does ADAM17 deplete ACE2 in mouse islets during diabetes progression, rather only regulates its shedding from the cell membrane.^51^

## 4 ROLE OF ACE2 IN BETA CELL DEVELOPMENT, FUNCTION, AND DIABETES

### 4.1 ACE2 and diabetes

Under pro‐inflammatory stress, such as IL‐1β+IFNγ and IFNα, but not fatty acid‐mediated lipotoxic conditions, expression of ACE2 mRNA is increased in human islets as well as in the human beta cell line EndoC‐βH1.^52^ This suggests that under diabetes‐related inflammatory stress, ACE2 expression increases. In line with this, ACE2, ADAM17 and TMPRSS2 expression is significantly increased in islets from diabetic individuals compared with non‐diabetics.^53^

**FIGURE 2:**
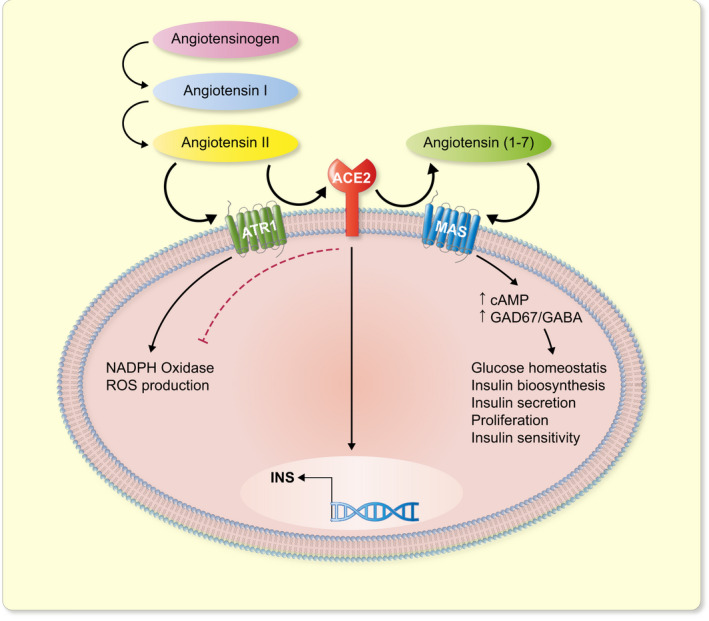
Schematic representation of the role and function of ACE2 and downstream molecules in pancreatic beta cells. Angiotensin II acting through the receptor AT1R, increases ROS production. Angiotensin II however gets metabolized to Angiotensin (1‐7) by ACE2. Angiotensin (1‐7) then, acting through its receptor MAS, downregulates NADPH oxidase activity and oxidative stress. Furthermore, Angiotensin II/ Angiotensin (1‐7)/ MAS signalling regulates pancreatic beta cell proliferation, insulin biosynthesis and secretion, thereby maintaining glucose homoeostasis

ACE2 gene dosage has drastic effects on glycaemic levels and beta cell function (Figure 2). C57BL/6 male mice deficient in *ACE2* gene fed with high‐fat diet demonstrate a lack of adaptive hyperinsulinemic response by beta cells towards high‐fat feeding (Table 1).^25^ Plasma insulin levels in these mice on standard diet are low and they further decrease when fed with high‐fat diet chronically.^25^ Interestingly, insulin expression in islets on ACE2‐knock out NOD mice is also decreased.^54^ Following 1 month of high‐fat feeding, the ACE2‐deficient mice demonstrates impaired GSIS in vivo.^25^ Interestingly, ACE2‐deficient mice on a low‐fat as well as high‐fat diet have a decreased beta cell mass and overall islet size. ACE2‐deficient mice again display a diminished adaptive response of increase in beta cell mass compared with normal mice, when both were fed a high‐fat diet.^25^ The HOMA‐β values indicating beta cell function are lower in ACE2‐deficient mice compared with the control mice when fed a high‐fat diet.^25^ These defects in GSIS due to ACE2 deficiency could not be restored by treatment with ATR1 antagonist or Ang (1‐7) peptide in vivo.^25^ Nonetheless, genetic augmentation of ACE2 in the pancreas of obese and diabetic mice leads to improved glucose tolerance as well as increased islet insulin content. It also increases beta cell proliferation and reduced beta cell apoptosis (Table 1).^55^ Additionally, *ACE2*‐KO mice showed an increase in the dedifferentiation of beta cells as indicated by the co‐expression of Insulin and Octamer‐binding transcription factor‐4 (Oct4), which is exaggerated further by high‐fat diet.^34^ However, administration of Ang (1‐7) peptide significantly ameliorates the dedifferentiation of beta cells under high‐fat diet conditions through improvement in islet microcirculation, which results from an upregulation of VEGF expression and reduction of increased islet iNOS activity due to high‐fat diet (Table 1).^34^

Ang (1‐7), on the other hand, improves insulin sensitivity, insulin biosynthesis, and beta cell survival in streptozotocin (STZ)‐induced T2D in rats.^56^ Ang (1‐7) downregulates diabetes‐induced iNOS, caspase family members like cleaved caspase 3, cleaved caspase 8 and cleaved caspase 9 as well as Bax expression in the rat pancreas, which are key genes involved in cellular stress and apoptosis, aggravating diabetes progression.^56^ Ang (1‐7), acting through its receptor MAS, improves insulin secretion by increasing cAMP production (Figure 2).^57^ Genetic Mas receptor ablation or its pharmacological blockade both result in diminished insulin secretion in response to glucose as well as depolarizing agents, such as potassium chloride (KCl)^57^ (Table 1). In a different study, ACE inhibitors (ACEIs) reversed insulin resistance as well as ROS production and attenuated glucotoxicity in isolated human islets, indicating that downregulating RAS activity on the other hand has beneficial effects on glucose homoeostasis.^58^

Previous studies reported that treating patients with Angiotensin II receptor blockers (ARBs) and ACE inhibitors (ACEi) increases the expression and activity of ACE2, particularly in the cardiac cells, putting the tissue at risk for SARS‐CoV‐2 binding.^59^, ^60^ Studies have also suggested that patients with hypertension and diabetes are at a higher risk for SARS‐CoV‐2 infection, as they are treated with such drugs.^61^ However, recent data have suggested that the long‐term treatment with ACEIs and ARBs has no effect on increasing the risk of infection with SARS‐CoV‐2 or on the severity of COVID‐19.^62^, ^63^, ^64^ On the other hand, ACEi attenuated the increased expression of angiotensinogen, ACE and ATR1 due to high glucose, in addition to protecting human islets from glucotoxicity and enhanced ER stress under hyperglycaemic conditions.^65^ ARBs, such as Valsartan, can improve first‐phase glucose‐stimulated insulin secretion as well as insulin sensitivity in subjects with perturbed metabolism.^66^ This indicates that downregulating the Ang II/ATR1/ATR2 axis may improve beta cell function, by potentially augmenting the ACE2/Ang (1‐7)/ Mas pathway.

### 4.2 Can SARS‐CoV‐2 infect pancreatic islets and cause diabetes?

**FIGURE 3:**
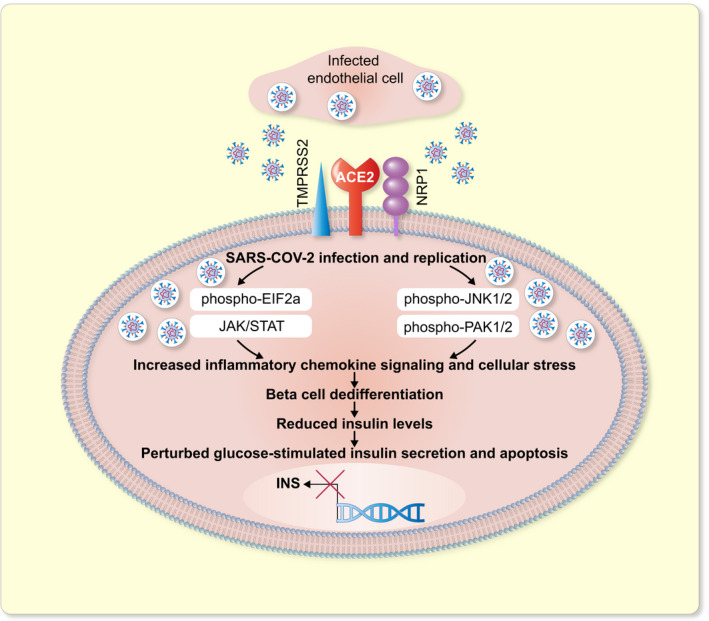
Graphical representation of how SARS‐CoV‐2 perturbs beta cell response upon direct invasion of pancreatic beta cells. SARS‐CoV‐2 travels to the pancreas through the infected endothelium, where it binds to the ACE2 and/or Neuropilin 1 (NRP1) receptors on pancreatic beta cells, infects and replicates within them. This leads to activation of the JAK/STAT, PAK1/2, JNK1/2, EIF2a pathways causing upregulation of stress and inflammation. Finally, beta cell dedifferentiation occurs wherein INSULIN levels are reduced, and beta cell loses its identity which severely impacts glucose‐stimulated insulin secretion, leading to an upregulation of cellular stress response and apoptosis

A recent study has shown that the ACE2 is expressed in the exocrine and endocrine pancreatic cells of normal human pancreas and its expression in the pancreas is higher than that in the lungs.^4^ Other studies reported that ACE2 is expressed in human pancreas, mainly in beta cells and in pancreas microvasculature or pericytes as well as in pancreatic ductal cells but with lower expression levels.^21^, ^26^ In frozen pancreatic sections, a strong ACE2 expression was seen in the endothelial cells and CK19‐positive ductal cells with a moderate expression in endocrine cells. Amongst the endocrine cell types, ACE2 and TMPRSS2 was most strongly expressed in C‐PEPTIDE‐positive cells.^21^ These findings indicate that SARS‐CoV‐2 may damage the pancreatic cells directly through its binding to ACE2 protein (Figure 3). However, other studies have presented contradictory findings suggesting that ACE2 is not highly expressed in the endocrine pancreas.^67^, ^68^ At least two studies have showed that ACE2 and TMPRSS2 protein are absent from pancreatic beta cells but rather expressed in the ductal epithelium of the pancreas and its microvasculature,^68^, ^69^ while other groups have demonstrated the low expression of ACE2 and TMPRSS2 in endocrine cells.^70^ Importantly, this variation in the expression of ACE2 and TMPRSS2 in human pancreata was explained by another study that described ACE2 to be weakly present in the islets of control individuals; however, in COVID‐19 patients, ACE2 was expressed in the beta cells of some of them, while it was only expressed in the fibroblasts for others.^22^ Interestingly, TMPRSS2 expression was observed by the group in islets only in the control individuals; however, it was found in both endocrine and exocrine pancreas in the deceased patients.^22^

Interestingly, one study showed that other newly discovered entry factors for SARS‐CoV‐2 such as NRP1 (Neuropilin 1)^71^, ^72^, ^73^, ^74^ and TFRC (Transferrin Receptor)^75^ are rather highly co‐expressed with beta cell marker, INSULIN, in healthy pancreata. The study found that while there was no stark difference in ACE2 and TMPRSS2 protein expressions between alpha and beta cells in the islet, both being low, NRP1 and TFRC were selectively expressed in the beta cells with a relatively high expression. Particularly, NRP1 expression was significantly higher in the pancreata of COVID‐19 deceased donors compared with healthy donors, while no significant difference was observed for ACE2 across infected or healthy donors.^70^ Few groups have also explored DPP4 (Dipeptidyl peptidase IV) or CD26 (Cluster of Differentiation 26) as candidate markers for SARS‐CoV‐2 entry into host cells. DPP4 is expressed in human islets, both in alpha and beta cells, as well as in the exocrine pancreas and several other tissues such as the adipose, epithelium and immune cells.^22^, ^76^, ^77^, ^78^ DPP4 was previously confirmed to be an entry factor for the MERS‐COV virus as well as the human coronavirus‐EMS.^79^, ^80^ Computational models have predicted protein structures of SARS‐CoV‐2 spike protein bound to DPP4 epitope to determine the effectiveness of their interaction.^81^ While the overall bound domains were similar in their structure to the MERS‐COV‐DPP4 complex, crucial differences in amino acid sequence in the binding domain of the two viruses render the SARS‐CoV‐2‐DPP4 binding weak.^82^ Additionally, in functional assays that purified DPP4 failed to bind the SARS‐CoV‐2 spike protein.^83^ Therefore, further investigations through autopsies and studies on isolated human islets are yet to validate a prominent SARS‐CoV‐2‐DPP4 interaction that facilitates its entry. Furthermore, soluble form of DPP4 (sDPP4) was found to be decreased in COVID‐19 patients,^84^ potentially making them susceptible to infection as it was shown that sDPP4 in the bloodstream could bind and sequester the virus and prevent it from attacking host cells.^85^ Nevertheless, evidence hints at a plausible role played by DPP4 in SARS‐CoV‐2 internalization. Of note, DPP4 inhibitors that lower DPP4 activity have widely been used to improve post‐prandial insulin levels in T2D patients and beneficial effects on clinical outcome was reported by some studies on COVID‐19 patients with T2D that continued DPP4 inhibitor regimen.^86^, ^87^ Another marker, Basigin or CD147 (Cluster of Differentiation 147) was pursued as a SARS‐CoV‐2 candidate entry factor, however, deleting CD147 from the surface of human lung cells yielded no differences in the level of infection.^88^, ^89^, ^90^ Therefore, SARS‐CoV‐2 spike protein entry into host cells in humans may be synchronized by co‐ordination with multiple surface receptors, in addition to or in the absence of ACE2, which remain to be validated.

Notably, cases of pancreatitis in COVID‐19 patients have been reported wherein patients present abdominal pain and elevated serum amylase or lipase, indicating exocrine pancreatic injury.^6^, ^7^, ^91^ It is unclear whether the associated pancreatitis is due to organ dysfunction owing to the cytokine storm resulting from SARS‐CoV‐2 infection or due to direct invasion of the virus in the pancreatic cells.

Nevertheless, it has been demonstrated that SARS‐CoV‐2 infects human islets both in vitro and in vivo. Isolated human pancreatic islets transfected with SARS‐CoV‐2 ex vivo showed the presence of viral spike proteins in the infected islets.^21^, ^70^, ^92^ Specifically, C‐PEPTIDE‐positive cells showed expression of viral S and N proteins; however, GLUCAGON and SOMATOSTATIN‐expressing cells did not, thus providing evidence that SARS‐CoV‐2 specifically infects pancreatic beta cells in the islet.^21^, ^70^ Furthermore, most of the S^+^/N^+^ islet cells had lost the expression of endocrine hormones but expressed the marker NKX6.1, which is restricted to beta cells amongst other endocrine cells.^21^, ^70^ These results indicate dedifferentiation of beta cells following SARS‐CoV‐2 infection to a non‐functional, immature progenitor state (Figure 3). Thus, another possible mechanism by which SARS‐CoV‐2 can cause diabetes is by inducing pancreatic beta cells to lose their identity upon direct infection. However, what downstream processes post SARS‐CoV‐2 infection cause beta cell dedifferentiation or degranulation is yet to be determined; however, it is hypothesized that cytokine storm and resultant ER stress pathways may be at play.

Nevertheless, Müller et al further provided evidence that SARS‐CoV‐2 infects human pancreatic cells in COVID‐19 patients by demonstrating viral N protein expression in pancreatic sections of COVID‐19 patients.^21^ Importantly, they found a rare and weak co‐expression of N protein with INSULIN^+^ cells in the islets of COVID‐19 patients; however, SARS‐CoV‐2 infection was strongly present in the vicinity of the islets.^21^ These findings are in line with those of Kusmartseva et al as the group could not show a co‐localization of INSULIN with viral proteins in the pancreatic tissues of COVID‐19 patients (Figure 3).^68^ Furthermore, Müller et al analysed other beta cell markers such as NKX6.1, which is exclusive to beta cell lineage in the adult pancreas and found that viral N^+^/NKX6.1^+^ cells were abundantly found in the pancreata of all four COVID‐19 patients investigated.^21^ Absence of N^+^/INSULIN^+^ in conjunction with the presence of N^+^/NKX6.1^+^ in the abovementioned samples, in addition to the decrease in insulin granules, validates the suggested beta cell degranulation upon infection in ex vivo cultured human islets (Figure 3). Interestingly, the infection of pancreatic beta cells by SARS‐CoV‐2 could be reversed to some degree ex vivo by remdesivir.^21^

On the other hand, Wu et al showed the co‐expression of INSULIN with the SARS‐CoV‐2 nucleocapsid protein in the islets of deceased patients.^70^ Notably, they observed a decrease in insulin expression as well as glucose‐stimulated insulin secretion of the ex vivo‐infected human pancreatic islets. The group also observed an increase in apoptotic beta cells in the islets upon infection; all of which were reversed using a NRP1 antagonist indicating that NRP1 is an entry factor that the virus utilizes to invade beta cells.^70^ They also showed that the viral binding to its receptors is enough to trigger apoptosis in pancreatic beta cells prior to the contribution of downstream stress pathways that are upregulated due to viral infection and replication by demonstrating the increases in phosphorylation of JNK1/2 and PAK1/2 upon treatment with just the SARS‐CoV‐2 spike protein.^70^

Also, another group has been recently able to demonstrate SARS‐N (nucleocapsid) immunopositivity in INS+/E‐Cadherin+beta cells along with alpha and delta cells, exocrine, endothelial and mesenchymal cells around the islets.^93^ Single‐cell sequencing also identified SARS‐CoV‐2 infection in INS+beta cells and highlighted the affected biological pathways in the infected islets which included interferon, cellular stress response, eukaryotic translation initiation factor 2 (EIF2a) and JAK‐STAT signalling pathways. In addition, chemokines and cytokine production by infected islets was significantly increased. Upon infection, insulin transcript levels were reduced with a concomitant increase in alpha cell markers such as GCG, SMARCA1, RGS4, KLHL41, RFX6, TM4S4 and acinar cell markers such as PRSS1, PRSS2, SPINK1, CPB1, CPA1, CPA2, OLFM4. The proportion of INS+/GCG+endocrine cells that were polyhormonal was high in infected islets indicating a trend towards transdifferentiation of beta cells. Phosphorylated levels of PKR and EIF2a were increased in infected islets along with the formation of stress granules. One chemical inhibitor of EIF2a signalling, Trans‐ISRIB (trans‐integrated stress response inhibitor), was found to prevent beta cell transdifferentiation in hPSC‐derived endocrine cells and prevent infection of human islets by SARS‐CoV‐2.^93^

Given the above advances in our understanding of how SARS‐CoV‐2 infects pancreatic islets and its consequences, it is important to highlight that due to fast viral evolution leading to multiple strains that differ in their infectious nature, we must assess the affinity of each strain to the different entry factors such as ACE2, TRFC, NRP1, DPP4, CD147 and others on pancreatic beta cells. Nonetheless, in line with these findings, previous studies showed that pancreas of patients infected with SARS‐COV stains positive for the SARS‐COV, indicating that the virus could invade pancreatic cells and could cause diabetes (Figure 3).^94^ Furthermore, SARS‐CoV‐2 and SARS‐CoV have high genetic similarity and both viruses bind the same surface protein, ACE2, to invade the target cells.^95^

Using a human pluripotent stem cell (hPSC)‐based model, following infection with SARS‐CoV‐2, the hPSC‐derived pancreatic endocrine cells showed a substantial proportion of spike protein‐positive SARS‐S^+^ INS^+^ and SARS‐S^+^ GCG^+^ cells.^92^ This indicates that SARS‐COv‐2 is able to infect both alpha and beta cells in the islet and therefore could directly control the development of diabetes in the COVID‐19 patients. Upon infection with SARS‐CoV‐2, insulin resistance pathway as well as the expression of inflammatory cytokines and chemokines, such as Interleukins, CCL‐ and CXCL‐family, and CASPASE 3 increase in hPSC‐derived pancreatic endocrine cells.^92^ However, it is yet to be investigated how SARS‐CoV‐2 could reach the pancreas from the lung remains a rather fascinating research avenue.

What course SARS‐CoV‐2 takes to reach the pancreas is unclear, but evidence hints at its replication and release from the infected cells. Interestingly, viral shedding through the gastro‐intestinal tract was evident when rectal swabs of children tested positive for viral RNA.^96^ SARS‐CoV‐2 virions can continuously escape the infected cells as reported in a study on human bronchial epithelial cell line (16HBE).^97^ Therefore, it is plausible that following SARS‐CoV‐2 infection in the lung cells, the virus is able to invade other tissue‐types, such as the pancreas through infected cells in the blood, for example, the T cells (Figure 3). While multiple studies have hinted at such possibility, it is yet to be demonstrated in vitro and in vivo. To this end, it is known that SARS‐CoV‐2 is able to directly infect secondary lymphoid organs, such as human lymph nodes and spleen.^98^ Additionally, an important study highlighted that SARS‐CoV‐2 could not only infect hPSC‐derived blood vessel organoids but also the infected organoid cells could produce viral progeny capable of infecting other cell types.^99^ Nonetheless, T cells and macrophages are permissive to SARS‐CoV‐2 and upon infection become glycolytic thus facilitating its replication.^100^ Higher glucose levels further enhance the rate of infection and replication of the virus, thereby hinting at the likelihood that hyperglycaemic patients may be at a higher risk of SARS‐CoV‐2 infection of different organs and experience more severe form of the disease.^100^ SARS‐CoV‐2 infection causes upregulation of PD‐1 that curbs T lymphocyte proliferation in vitro indicating a weaker immune response.^100^ However, T lymphocytes and macrophages can infiltrate pancreatic islets and lead to beta cell destruction.^101^ Further studies are, nevertheless, needed to investigate how SARS‐CoV‐2 travels to the islets.

Apart from direct infection, multiple indirect routes for development of diabetes in a COVID‐19 patient should be considered. One reason could be the harmful influence of immune reaction caused by the SARS‐CoV‐2 infection that could affect beta cell function and survival due to islet inflammation.^102^, ^103^ The surge in secretion of IL‐6 (Interleukin 6) and IL‐10 (Interleukin 10) as well as other inflammatory cytokines upon SARS‐CoV‐2 infection, also referred to as ‘cytokine storm’, that can decapitate organs and cause apoptosis.^104^ Strikingly, IL‐6 levels are increased in T2D patients infected with SARS‐CoV‐2 explaining why those with pre‐existing diabetes suffer from a more severe form of inflammatory stress which can further compromise islet function and cause beta cell destruction along with pancreatitis or even multi‐organ failure.^105^ Also, exocrine pancreas injury as reported in multiple COVID‐19 patients may also lead to islet casualty resulting in hyperglycaemic conditions. Therefore, a direct molecular link between SARS‐CoV‐2 and diabetes is yet to be deciphered. Recently, an international registry (COVIDiab) has been established to understand the relationship between COVID‐19 and diabetes.^106^

## 5 CONCLUDING REMARKS

Given the ability of SARS‐CoV‐2 to infect the pancreas in vivo and ex vivo and the crucial role of ACE2 in regulating pancreatic beta cell function, it is presumed that the effect of SARS‐CoV‐2 infection on diabetes pathogenesis is mediated, in part at least, through ACE2 direct binding in the pancreas. However, with multiple studies showing differences in the levels of ACE2 expression within the pancreas, and the demonstration of other receptors such as NRP1 as an entry factor for SARS‐CoV‐2 in the islet suggests that ACE2 may coordinate with other potential entry factors in facilitating SARS‐coV‐2 invasion in the islets. Importantly, in vitro and in vivo investigations have demonstrated SARS‐CoV‐2 presence in the pancreas of COVID19 patients, and its replication in the infected islets and shedding has also been demonstrated. Since the virus can also infect the endothelium and monocytes, it is likely that in cases of severe infection the virus can travel to the islets through the blood (Figure 3). Nevertheless, how the virus causes diabetes following islet infection is yet to be investigated; however, loss of a beta cell identity through dedifferentiation, degranulation, and upregulation of inflammatory stress is a potential underlying mechanism. While the majority of our understanding of ACE2 function in the pancreas is determined from rodent studies, in addition to immortalized cell lines, both of which cannot accurately capture the key protein–protein interactions during SARS‐CoV‐2 binding to the host and cellular host responses to viral replication in humans. In addition, there are differences in the expression levels of ACE2 receptor amongst the different endocrine cells in the islets in rats, mice, and humans.^4^, ^35^, ^107^, ^108^ Therefore, majority of the existing knowledge on ACE2 function in the pancreas cannot be extrapolated to humans. The recently established hPSC‐derived pancreatic organoids and beta cells^92^, ^109^ could serve as a brilliant platform in evaluating the role of ACE2 in regulating insulin secretion as well as on how ACE2 function in the beta cells is affected following SARS‐CoV‐2 and its implications on diabetes pathophysiology, thus warranting extensive studies.

## CONFLICT OF INTERESTS

The authors declare that they have no competing interests.

## AUTHOR CONTRIBUTIONS

Memon B and Abdelalim EM discussed the concept of the review, worked on the outline, and wrote the review. Both authors critically reviewed the manuscript and approved the final version for submission.
